# Autism and intellectual disability due to a novel gain-of-function mutation in *UBE3A*

**DOI:** 10.1038/s10038-025-01343-z

**Published:** 2025-05-02

**Authors:** Anna M. Gunelson, Kwang-Soo Kim, Connolly G. Steigerwald, Devorah Segal, Nicolas J. Abreu, Jason J. Yi

**Affiliations:** 1 Department of Neuroscience, Washington University School of Medicine, St. Louis, MO 63110 USA; 2 Department of Neurology, NYU Grossman School of Medicine, New York, NY 10016 USA

**Keywords:** Autism spectrum disorders, Genetics of the nervous system

## Abstract

The loss of maternal *UBE3A* causes Angelman syndrome whereas its duplication is associated with a heterogeneous neurodevelopmental disorder. Here, we describe two affected brothers who possess a novel *UBE3A*^L734S^ variant that is not present in two neurotypical siblings. The *UBE3A*^L734S^ variant was confirmed to be maternally inherited, and the affected individuals exhibited early global developmental delay, ongoing learning difficulties, and autistic features. Their phenotypes were inconsistent with Angelman syndrome. Biochemical characterization showed the *UBE3A*^L734S^ variant causes a dramatic increase in the activity of the UBE3A enzyme, suggesting that a gain in UBE3A activity is the driver of neurodevelopmental disease. Our observations document an emerging class of neurodevelopmental disorders caused by gain-of-function mutations in *UBE3A*.

## Introduction

*UBE3A* is a gene that encodes a HECT (homologous to E6AP C-terminus) domain E3 ubiquitin ligase that targets substrate proteins for degradation through the ubiquitin-proteasome pathway [1]. *UBE3A* is located on chromosome 15q11-13 and its expression is imprinted in neurons such that the paternal gene is epigenetically silenced and only the maternal gene is expressed [2–5]. UBE3A is widely associated with Angelman syndrome, which is caused by deletion or mutation of maternal *UBE3A* [3, 6]. In contrast, duplication or triplication of maternal chromosome 15q11-13, the chromosomal region where *UBE3A* resides, causes a prevalent genetic form of autism known as Dup15q syndrome [7].

In previous work, we characterized 152 variants of uncertain significance in *UBE3A* and found that ~12% of non-truncating variants in *UBE3A* represent gain-of-function mutations [8]. Individuals possessing these mutations in maternal *UBE3A* presented with varying levels of intellectual disability, autistic features, motor deficits, skeletal abnormalities, and a partial penetrance for seizures [8]. However, detailed genotype and phenotype information about such patients remains poorly documented.

### Case report

**Fig. 1:**
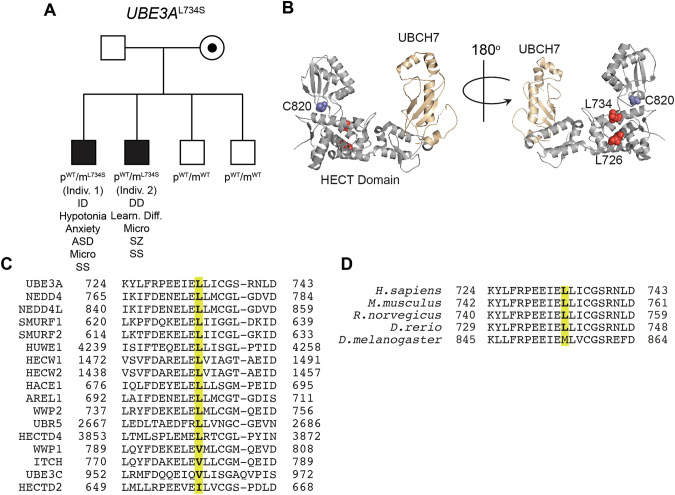
*UBE3A*^L734S^ segregates with neurodevelopmental phenotypes. **A** Pedigree of a family showing maternal inheritance of the *UBE3A*^L734S^ variant; intellectual disability (ID); developmental delay (DD), autism spectrum disorder features (ASD), learning difficulties (Learn. Diff.), microcephaly (Micro), schizophrenia (SZ), short stature (SS). **B** Crystal structure (PDB: 1C4Z) of the HECT domain of UBE3A (gray) bound to the E2 ubiquitin conjugating enzyme UBCH7 (gold). Catalytic cysteine (C820) in blue, L734 and L726 in red. **C** Sequence alignment showing conservation of L734 in UBE3A and surrounding amino acids across HECT domain family enzymes. **D** Sequence alignment showing the high degree of evolutionary conservation of L734 of UBE3A and surrounding amino acids across indicated species

We identified two siblings possessing a missense variant that substituted a serine at leucine 734 (*UBE3A*^L734S^) in UBE3A, *UBE3A*(NM_130838.1):c.2201T>C p.Leu734Ser. This mutation was confirmed to be maternally inherited and present in two affected brothers and absent in two neurotypical brothers (Fig. 1A). No other disease-causing variants were identified in exome or microarray analysis. Individual 1 was 13 years of age at the time of initial neurogenetics evaluation for developmental delay. He possessed short stature (Z = −3.99 on CDC curves), limited expressive language composed of short phrases, a long nose, deep-set eyes, retrognathia, upper back kyphosis, flesh-colored papules on his knuckles, chronic thrombocytosis, and borderline microcephaly (Z = −1.75 on Nellhaus curves). Early development was notable for first words by 14 months and independent ambulation at 20 months old. He developed intellectual disability, hypotonia, anxiety with autistic features, strabismus, and no seizures. Individual 2 was 21 years old at the time of examination. There were early developmental delays with his first words by 19 months of age and independent ambulation at 2 years old. He possessed short stature (Z = −3.78 on CDC curves), ptosis, retrognathia, cervical kyphosis with lumbar lordosis, ventricular septal defect surgically repaired in infancy, cool/red hands, lower extremity hyperreflexia, and borderline microcephaly (Z = −1.55 on Nellhaus curves). He spoke in sentences. He had a history of learning difficulties, mild inattention, strabismus, and no seizures. He developed schizophrenia after the initial evaluation. The brothers did not possess ataxic gait, tremor, or unprovoked laughter typically seen in patients with Angelman syndrome. Their mother was healthy, and received academic support briefly until high school. No one else in the family possessed short stature. Exome and microarray testing (GeneDx, Gaithersburg, MD) was otherwise non-diagnostic.

### Functional variant analysis

L734 is a residue within the catalytic HECT domain of UBE3A that houses all the necessary machinery to facilitate substrate ubiquitination [9]. Although L734 is not located near the catalytic cysteine of UBE3A (C820), it is in close proximity to a leucine at position 726 whose in-frame deletion was identified to cause a gain-of-function of UBE3A (*UBE3A*^L726Δ^; Fig. 1B) [8]. Sequence alignments showed the L734 is well conserved across species and across most HECT domain family enzymes (Fig. 1C, D), suggesting it may have a critical role in the ubiquitination process.

**Fig. 2:**
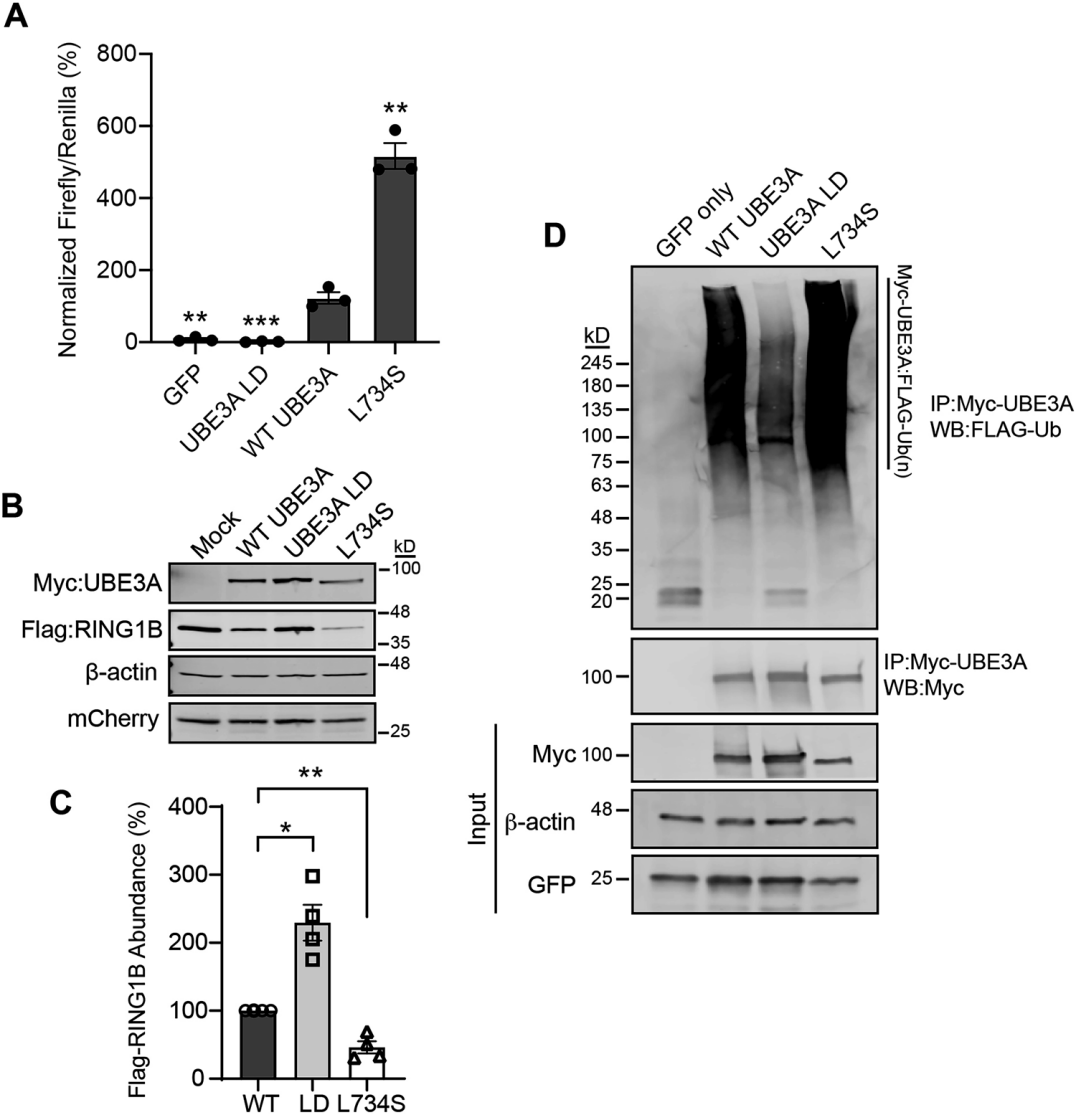
*UBE3A*^L734S^ is a gain-of-function mutation. **A** Cultured HEK293T cells were transfected with plasmids encoding GFP, UBE3A Ligase Dead (LD), WT UBE3A, or UBE3A^L734S^. BAR responses of variants were normalized to WT UBE3A responses in each experiment. *N* = 3 independent experiments. For each experiment, each variant was tested in triplicate and the resulting values averaged. Values are shown as the mean ± SE. The *p*-values were calculated using a one-sample t-test (two-tailed) with Benjamini–Hochberg multiple comparisons correction (FDR = 0.05). **p < 0.005, ***p < 0.0005. Representative western blot (**B**) and quantification (**C**) of protein lysates from HEK293T cells that were co-transfected with WT UBE3A, UBE3A LD, or UBE3A^L734S^ and Flag-RING1B, with mCherry as a transfection and loading control. Values in (**C**) are normalized to UBE3A protein levels in the WT transfection condition and are shown as the mean percent ±SE of FLAG-RING1B levels. *N* = 4, *p < 0.05, **p < 0.01, One-sample t-test (two-tailed). Representative images in (**B**) are shown from four  independent experiments that produced similar results. Original blots are presented in Supplementary Fig. 1. **D** Representative western blot of HEK293T cells transfected with Flag-ubiquitin and either GFP, WT Myc-UBE3A, Myc-UBE3A-LD (Ligase Dead), or Myc-UBE3A^L743S^ plasmid constructs. Cells were then treated with the proteasome inhibitor MG-132 (30μm) for 1 h. Ubiquitinated UBE3A was immunoprecipitated (IP) using c-myc conjugated magnetic beads and ubiquitination was probed by western blot analysis (WB) using an anti-Flag antibody to detect ubiquitinated UBE3A. Representative images are shown from four independent experiments that produced similar results. Original blots are presented in Supplementary Fig. 2

Using a previously developed luciferase-based UBE3A reporter system [8, 10], we found this variant increased enzyme activity roughly 500% above wild type (WT) levels (Fig. 2A). We biochemically validated *UBE3A*^L734S^ using its overexpression in HEK293T cells. These experiments demonstrated the *UBE3A*^L734S^ mutation was more efficient than WT UBE3A at reducing the steady-state levels of the UBE3A substrate RING1B [11] (Fig. 2B, C). Moreover, we found this variant enhanced its own self-targeted ubiquitination (Fig. 2D), confirming *UBE3A*^L734S^ as a gain-of-function mutation.

## Discussion

Our study found that maternal inheritance of the *UBE3A*^L734S^ variant segregates with phenotypes in siblings, but we could not confirm the parental origin of the *UBE3A*^L734S^ variant in the unaffected mother. However, we note that virtually all individuals with gain-of-function mutations in *UBE3A* possess maternally inherited mutations [8], suggesting that paternal inheritance may confer a significantly reduced risk for neurodevelopmental dysfunction. The siblings possessing the *UBE3A*^L734S^ mutation did not present with seizures and Individual 2 developed schizophrenia, both of which diverge from phenotypes in individuals with supernumerary copies of maternal chromosome 15q11-13. However, these phenotypes were similar to individuals with a maternal microduplication encompassing only *UBE3A* who do not present with seizures and have a family history of neuropsychiatric disorders including schizophrenia [12]. These observations suggest that increased UBE3A ubiquitin ligase activity alone may produce phenotypes that may overlap with, but also differ from supernumerary 15q11-13.

Our study does not elucidate the mechanisms by which the *UBE3A*^L734S^ mutation causes enzyme gain-of-function. However, we found the L734 residue is highly conserved, suggesting this residue plays a critical role in the catalytic cycle of HECT domain ubiquitin ligases. Future work will be required to understand how the L734 residue contributes to enzyme function, and whether orthologous mutations in other HECT domain enzymes lead to human disorders.

Finally, our results also highlight the importance of integrating functional variant analysis in medicine. Clinical trials using antisense oligonucleotides (ASOs) for the treatment of Angelman syndrome are currently underway [13–15]. As these reagents unsilence the expression of paternal *UBE3A*, they act to increase overall UBE3A activity in neurons. Our study demonstrates that some disease-causing mutations in *UBE3A* lead to neurodevelopmental pathology due to a gain in UBE3A activity. These results argue that understanding the functional impact of mutations in *UBE3A* is critical to distinguish sub-classes of *UBE3A*-dependent disorders and such knowledge will be necessary for the safe administration of *UBE3A*-targeting therapies.

## Methods

Detailed experimental methods are provided in the accompanying Supplementary Information.

## Supplementary information

Supplementary Information

Supplementary Table 1
