# Supplementary material for: Autism and intellectual disability due to a novel gain-of-function mutation in UBE3A
Source: J Hum Genet. 2025 May 2;70(8):439–42. doi: 10.1038/s10038-025-01343-z (PMC12289519; doi:10.1038/s10038-025-01343-z)
Supplement: Supplementary file 1 — Supplementary Information [file 10038_2025_1343_MOESM1_ESM.pdf]

Supplementary Information

Autism and intellectual disability due to a novel gain-of-function mutation in *UBE3A*

Anna M. Gunelson[1], Kwang-Soo Kim[1], Connolly G. Steigerwald[2], Devorah Segal[2], Nicolas J. Abreu[2], Jason J. Yi[1*]

[1]Department of Neuroscience, Washington University School of Medicine, St. Louis, MO, 63110, USA.

[2]Department of Neurology, NYU Grossman School of Medicine, New York, NY, 10016, USA

*Corresponding author: jasonyi@wustl.edu

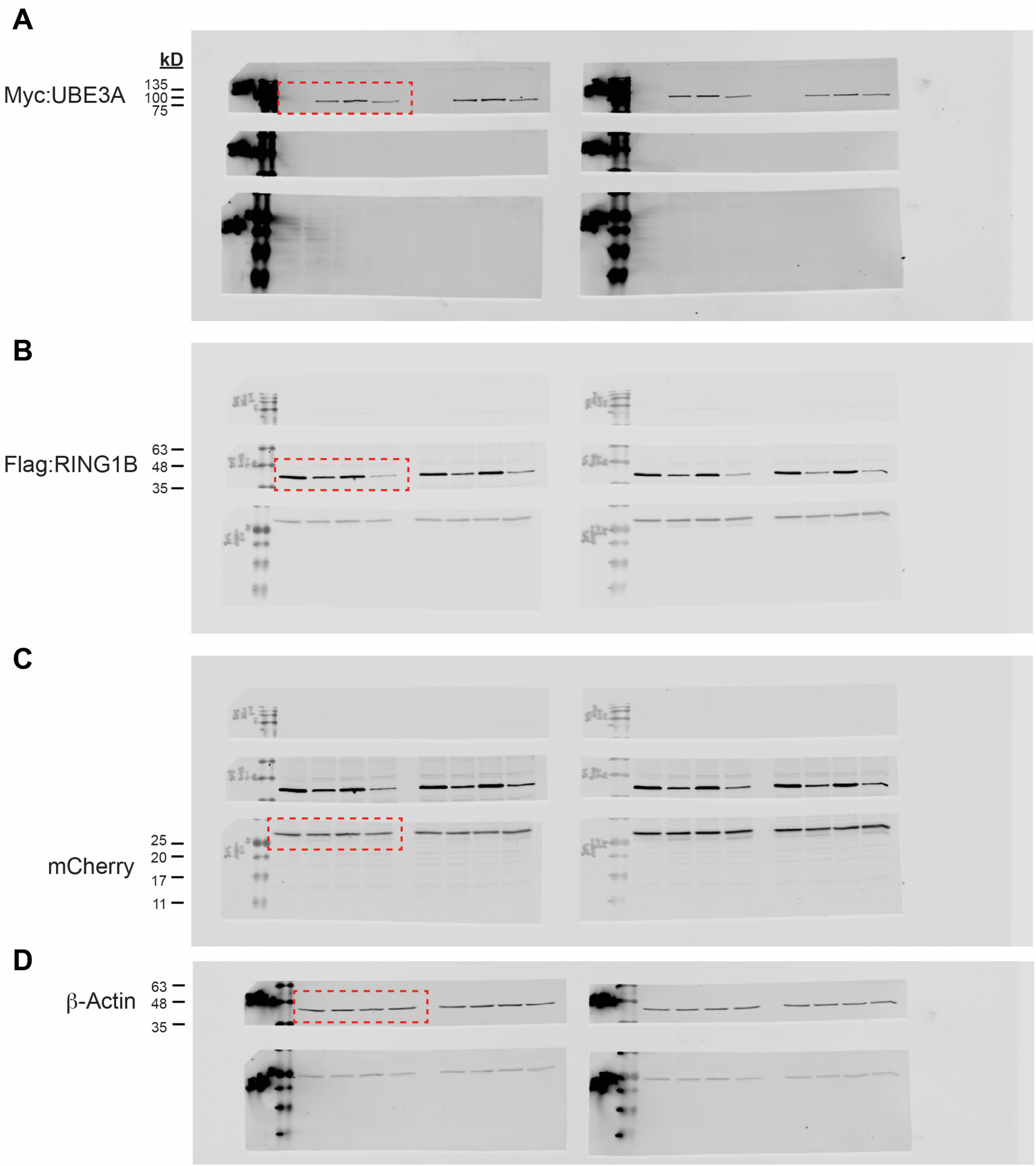

Supplementary Figure 1: Original western blot images for Fig. 2B.

Original western blot images used in Fig. 2B showing steady-state levels of RING1B in the presence of UBE3A. The original western blot image contains two replicates. Red box shows where the image was cropped for the figure. Gels were transferred on to a single membrane and the resulting membrane was cut to blot individually for: A) Myc:UBE3A (~100 kD); B) FLAG:RING1B (~45 kD); C) mCherry (~27 kD); and D) β-actin (~42 kD). Note a longer exposure was used to visualize mCherry bands compared to FLAG:RING1B.

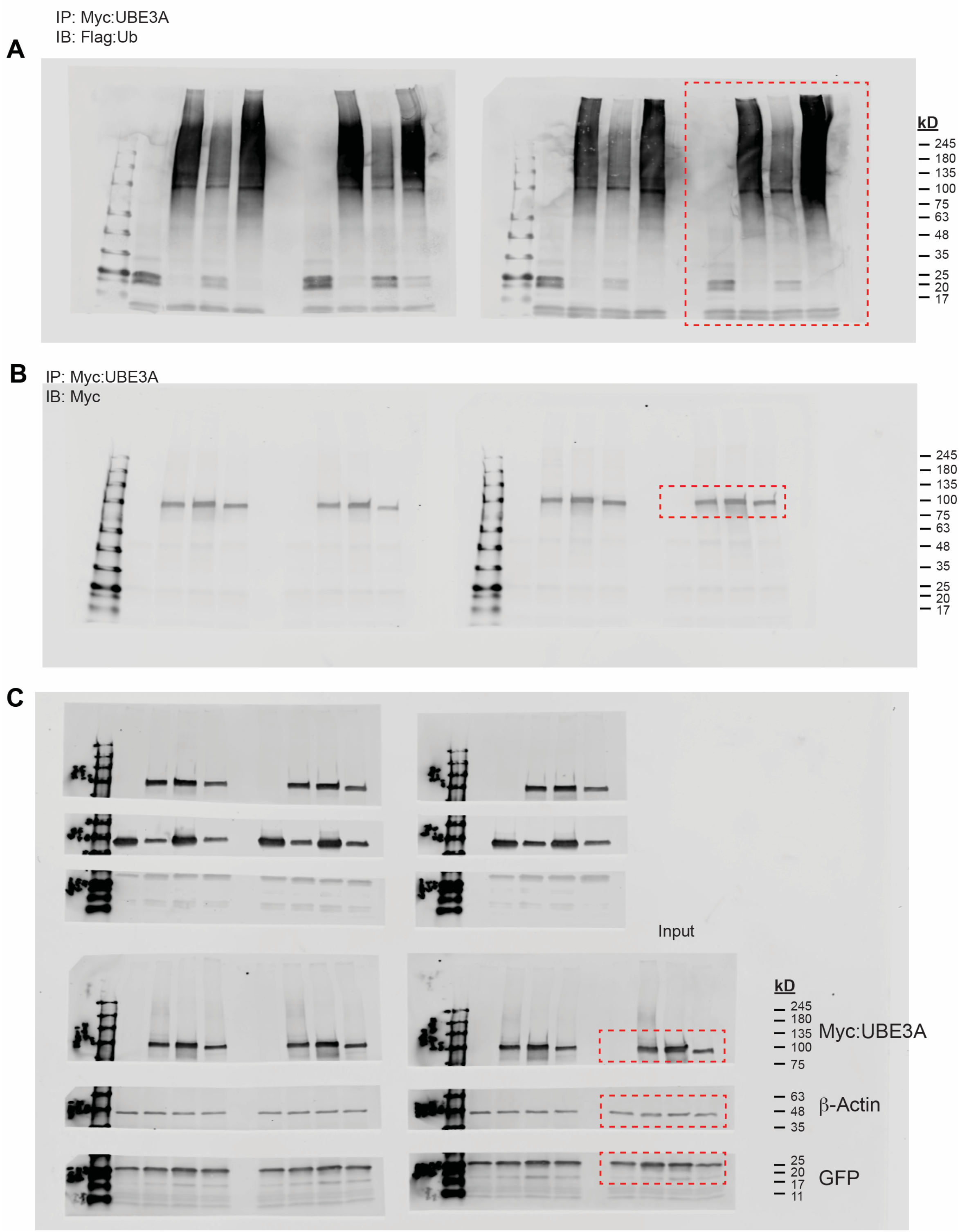

Supplementary Methods Supplementary Figure 2: Original western blot images for Fig. 2D.

A and B) Original western blot images used for immunoprecipitation experiments shown in Fig. 2D. The original western blot image contains four replicates. Red box shows where the image for the figure was cropped. C) Gels for input blots were transferred on to a single membrane and the resulting membrane was cut to blot individually to probe for Myc:UBE3A (~100 kD), β-actin (~42 kD), and GFP (~27 kD). Red box shows where the image for the figure was cropped.

Supplementary Methods

Cell Culture and Transfection

HEK293T cells (ATCC) were maintained in a 5% CO_2 humidified incubator in DMEM supplemented with 25 mM glucose, 0.4 mM glutamine, 1 mM Sodium Pyruvate (Gibco #10569044), 10% fetal bovine serum (Gibco, #16140071), and 1× antibiotic–antimycotic containing penicillin, streptomycin, and amphotericin B (ThermoFisher #15240062). For biochemical analyses, transfections were performed using FuGENE (Promega) according to the manufacturer's instructions.

Molecular Biology

The nomenclature used in this study was based on the amino acid sequence from human UBE3A isoform I (NCBI accession: NP_001361390.1). A detailed protocol to generate Myc-tagged variants of human UBE3A is published (1). In brief, Myc epitope tags were placed on the N-terminus of UBE3A by polymerase chain reaction and all UBE3A constructs were cloned into pCIG2 plasmid DNAusing SacI and XmaI sites.A FLAG-tagged, catalytically-inactive mutant of human RING1B (I53S) (8), pGL3-BAR and TK-Renilla luciferase (2), and pCDNA3.13 3x FLAG-tagged ubiquitin (3) were all described in previous studies.

Biochemistry

Ubiquitination assays were performed in HEK293T cells were transfected with the indicated constructs along with FLAG-tagged ubiquitin. After 48 hours (h), cells were treated with 30 µM of the proteasome inhibitor MG-132 (Sigma Aldrich, #474790) for 1 h. Cells were lysed in cell lysis buffer containing: 25Mm HEPES pH7.4, 1% Triton X-100, 15mM KCl, 5mM EDTA) containing 1% SDS and protease inhibitor (Thermo Fisher #A32955), boiled for 30 minutes (min) and clarified by centrifugation at 12,000 x g for 15 min. The resulting supernatant was diluted 1:10 (v/v) with cell lysis buffer without SDS. Lysates were pre-cleared using Dynabeads Protein G beads (Thermo Fisher #10003D) for 1 h at 4°C and UBE3A was immunoprecipitated using anti-c-Myc magnetic beads (Thermo Fisher #88842) for 18 h at 4°C. The final complex was washed twice in 1x TBST and once in

ultrapure water, resuspended in sample buffer (Thermo Fisher, #NP0008) supplemented with 5 mM DTT, and boiled for 5 min. Proteins were resolved by 4–20% SDS-PAGE and transferred to nitrocellulose membranes (Bio-Rad). Membranes were blocked in 1× fish gelatin blocking agent (Biotium #22010) and probed with the appropriate primary antibodies overnight at 4 °C or 2 h at RT. Protein bands were visualized using the Odyssey CLx infrared imaging system and Image Studio v5.2 software (LI-Cor Biosciences) and western blots were analyzed using ImageJ v2.1.0/1.53c. For RING1B steady-state assays, HEK293T cells were transfected with the indicated Myc-tagged UBE3A constructs, FLAG-tagged RING1B, and pmCherry-N1. After 48 h, cells were lysed in cell lysis buffer, processed for western blot, and band intensities quantified.

A detailed protocol for the BAR luciferase assay to assess UBE3A activity was published in a previous study (1). In brief, HEK293T cells were plated at a density of 10,000 cells/well in a 96-well format. Cells were transfected with pRL-TK-Renilla, BAR-pGL3, and the indicated constructs using FuGENE transfection reagent. Forty-eight hours after transfection, reporter gene expression was assessed using the Dual-Luciferase reporter assay system (Promega) and measured on a Synergy HTX Multi-Mode Reader (BioTek) using Gen5 software v3.08. Luciferase activity was normalized against *Renilla* activity, and UBE3A missense variant signals were normalized to WT UBE3A.

Sequence alignments of UBE3A with various human HECT domain enzymes were performed using amino acid sequences with the following accession numbers: UBE3A (NP_001341442.1), NEDD4 (NP_006145.2), NEDD4L (NP_001138439.1), SMURF1 (NP_065162.1), SMURF2 (NP_065162.1), HUWE1 (NP_113584.3), HECW1 (NP_055867.3), HECW2 (NP_065811.1), HACE1 (NP_065822.2), AREL1 (NP_001034568.1), WWP2 (AAC51325.1), UBR5 (NP_056986.2), HECTD4 (NP_001103132.4), WWP1 (NP_008944.1), ITCH (Q96J02.2), UBE3C (NP_055486.2), HECTD2 (NP_877497.4). Sequence alignments of UBE3A from various species were performed using the following: *H. sapiens* (NP_001341442.1), *M. musculus* (NP_001029134.1), *R. norvegicus* (NP_001178766.1), *D. rerio* (NP_001007319.1), and *D. melanogaster* (NP_648452.1).

References

1. Stelzer JA, Yi JJ. A Scalable, Cell-based Method for the Functional Assessment of Ube3a Variants. J Vis Exp. 2022(188).

2. Yi JJ, Paranjape SR, Walker MP, Choudhury R, Wolter JM, Fragola G, et al. The autism-linked UBE3A T485A mutant E3 ubiquitin ligase activates the Wnt/beta-catenin pathway by inhibiting the proteasome. J Biol Chem. 2017;292(30):12503-15.

3. Weston KP, Gao X, Zhao J, Kim KS, Maloney SE, Gotoff J, et al. Identification of disease-linked hyperactivating mutations in UBE3A through large-scale functional variant analysis. Nat Commun. 2021;12(1):6809.
